# Gender dimorphic M1 excitability during emotional processing: a transcranial magnetic stimulation study

**DOI:** 10.7717/peerj.13987

**Published:** 2022-08-30

**Authors:** Fanghui Qiu, Yu Zhou, Lanlan Zhang, Jian Zhang, Hui Liu

**Affiliations:** 1 Department of Physical Education, Qingdao University, Qingdao, China; 2 Department of Physiology and Pathophysiology, School of Basic Medical Sciences, Qingdao University, China; 3 Department of Leisure Sports and Management, Guangzhou Sport University, Guangzhou, China; 4 School of Psychology, Shanghai University of Sport, Shanghai, China; 5 Shanghai Punan Hospital of Pudong New District, Shanghai, China

**Keywords:** Motor excitability, Gender, Emotion perception, Transcranial magnetic stimulation

## Abstract

**Background:**

It is widely held that emotions prime the body for action. However, the influence of gender on primary motor cortex (M1) excitability during emotional processing is not well explored.

**Methods:**

Using single-pulse transcranial magnetic stimulation (TMS), we stimulated the right or left M1 at 150 ms and 300 ms after emotional stimulation onset (presentation of negative, neutral, and positive pictures to male and female subjects). Motor-evoked potentials (MEPs) ratio induced by single-pulse TMS was used to assess changes in corticospinal excitability.

**Results:**

In response to right M1 stimulation, males demonstrated higher MEP ratios following presentation of negative pictures at 150 ms while MEP ratios in response to presentation of positive pictures were greater at 300 ms. Furthermore, male subjects showed larger MEP ratios in right M1 versus left M1 at 300 ms after initiation of positive pictures, indicating lateralization of motor excitability in male subjects.

**Conclusions:**

The current study thus provides neurophysiological evidence to support gender differences and functional lateralization of motor excitability in response to emotional stimuli.

## Introduction

The ability to quickly identify salient emotional information in the environment and to rapidly and appropriately respond, is critical to survival. Previous studies have reported gender differences in emotion processing. Specifically, women perform better than men on perception of negative emotions (Gard & Kring, 2007; Hamann, 2005; Yang et al., 2018), while men show a perceptual bias toward positive stimuli (Natale, Gur & Gur, 1983; Ran, 2018). To date, gender dimorphic studies on emotion processing have mainly focused on activity within emotion-related brain regions. However, it has been postulated that motor system is recruited during emotion processing, assuming that emotions prime the body for action (Borgomaneri, Gazzola & Avenanti, 2014; Eder & Rothermund, 2013; Hajcak et al., 2007; Nogueira-Campos et al., 2014). Prior studies have reported engagement of the motor cortex during emotion perception (Borgomaneri et al., 2015; Jabbi & Keysers, 2009). However, whether the motor cortex shows gender dimorphic excitability during emotion processing remains uncertain.

Transcranial magnetic stimulation (TMS) is a useful tool in the assessment of changes in corticospinal excitability (Hallett, 2007) and in the investigation of the interplay between action and emotion processing within the motor system (Borgomaneri, Gazzola & Avenanti, 2012; Borgomaneri, Gazzola & Avenanti, 2015). TMS studies have revealed a close relationship between action readiness and emotion, showing increased corticospinal motor excitability during emotion processing (Borgomaneri, Gazzola & Avenanti, 2012; Coelho et al., 2010; Hajcak et al., 2007; Komeilipoor et al., 2013; Loon et al., 2010; Onigata & Bunno, 2020). The above findings suggest that emotion perception may prime the motor system in order to facilitate action readiness.

Hemispheric lateralization of emotional processing has been extensively studied. Results have shown gender differences in the hemispheric lateralization of emotional experience (Cahill et al., 2004; Killgore & Yurgelun-Todd, 2001; Wager et al., 2003; Williams et al., 2005). For example, males exhibit greater lateralized activity in the right amygdala during processing emotional scenes (Cahill et al., 2004) and faces (Killgore & Yurgelun-Todd, 2001), whereas females show either left lateralization (Cahill et al., 2004) or minimal lateralization (Wager et al., 2003) in amygdala activity during emotional perception. Electrophysiological studies have revealed that emotional scenes and faces elicit a stronger P300 (Gasbarri et al., 2007) and occipito-temporal N1 component (Proverbio et al., 2006), respectively, in the right hemisphere in men. In contrast, women show a more robust P300 in the left hemisphere (Gasbarri et al., 2007) with little lateralization of the N1 response (Proverbio et al., 2006) when exposed to the same stimuli. These findings suggest gender dimorphic lateralization of cerebral cortex activation during emotional processing. However, studies have yet to investigate possible gender differences in hemispheric lateralization of the motor system during emotional processing.

We aimed to identify potential gender differences in hemispheric asymmetry in motor excitability during processing of emotional stimuli with different valences. We stimulated the right or left M1 at 150 ms and 300 ms after onset of emotional stimuli in separate sessions. The participants performed emotion recognition tasks, during which negative, neutral and positive pictures were presented. We hypothesized that males and females would differ in hemispheric lateralization of motor excitability during emotional stimulus processing of differing valence.

## Materials & Methods

### Participants

A total of 100 undergraduate students (50 males and 50 females) participated in the study. Fifty participants (25 males and 25 females) were randomly assigned to Group 1 in which the right M1 was stimulated, and the other 50 participants were assigned to Group 2 in which the left M1 was stimulated. All participants were free from contraindication to TMS (Rossi et al., 2009), right-handed, and reported normal or corrected-to-normal vision. None of the participants had a history of psychiatric or neurological disorder. None of the female participants were taking oral contraceptives or had a premenstrual syndrome. The experimental protocol was approved by the Ethics Committee at the Shanghai University of Sport (2017031). All participants provided written informed consent for participation prior to the start of the experiment. No discomfort or adverse effects of TMS were reported by participants.

### Stimuli

Twenty positive pictures (mean valence 7.39  ± 0.25, mean arousal 5.55  ± 0.53), 20 negative pictures (mean valence 2.08  ± 0.35, mean arousal 5.77  ± 0.78) and 20 neutral pictures (mean valence 5.03  ± 0.16, mean arousal 4.03  ± 0.86) were selected from the native Chinese Affective Picture System (Bai, Ma & Huang, 2005). Mean valence differed significantly (all *p* < 0.001), and the reported arousal in response to neutral pictures was significantly lower than that of positive and negative pictures (both *p* < 0.001).

### Transcranial magnetic stimulation

A single TMS pulse was applied over either the right (Group 1) or the left (Group 2) M1 with a figure-eight-shaped coil (outer diameter, 9.5 cm) connected to a Magstim 200 stimulator (Magstim, Whitland, Dyfed, UK). The handle of the coil pointed backward at approximately 45° from the mid-sagittal line. For each participant, the optimal position for activation where maximal amplitude motor-evoked potentials (MEPs) were elicited in the contralateral first dorsal interosseous (FDI) muscle was marked with a pen as the motor hot spot. Resting motor threshold (RMT) was defined as the lowest TMS intensity required to generate MEPs of more than 50 µV in at least five out of 10 consecutive pulses when the target muscle was completely relaxed. Single TMS pulses were delivered to the optimal scalp position with an intensity of 120% RMT (Xia et al., 2021). The absence of voluntary contractions was visually verified continuously throughout the experiment. When muscle tension was detected, the experiment was briefly paused and the participant was asked to relax.

### Electromyography (EMG) recording

EMG was recorded as previously described in Xia et al. (2021). Surface electromyograms were recorded from either the left (Group 1) or right (Group 2) first FDI muscle (contralateral to the stimulated hemisphere) with nine mm diameter Ag–AgCl surface electrodes. The active electrodes were placed over the muscle belly and the reference electrode over the metacarpophalangeal joint of the index finger. The signal was amplified (1,000×), bandpass filtered (20 Hz–2.5 kHz; Intronix Technologies Model 2024 F), digitized at 5 kHz by an analogue-to-digital interface (Micro1401; Cambridge Electronics Design, Cambridge, UK), and stored in a computer for off-line analysis.

### Procedure

The experimental protocol was programmed in and carried out by MATLAB software; the emotion recognition task was presented via Psychtoolbox for MATLAB and TMS was administered by the Data Acquisition Toolbox (DAQ) via DAQ code loaded in a Supplemental File. Before and after the experimental session, two blocks of 10 MEPs were collected using single-pulse TMS, which served as baselines. During these blocks, participants kept their eyes closed with the instruction to imagine watching a sunset at the beach and relax their hand muscles (Borgomaneri et al., 2015) while single-pulse TMS was administered with an inter-pulse interval of approximately 10 s.

The present study assessed motor excitability during emotional processing at both an early and a later time point. The early time point (150 ms) was chosen based on the finding that emotional pictures modulate visual event-related potentials (ERPs) within 100–200 ms (Olofsson et al., 2008) and change motor excitability within 150 ms (Borgomaneri, Gazzola & Avenanti, 2014; Borgomaneri, Gazzola & Avenanti, 2015). Early motor reactivity during processing of emotional stimuli reflects an adaptive response, readying the individual for fight/flight (Borgomaneri, Gazzola & Avenanti, 2015). The later time point (300 ms) was chosen based on the finding that motor excitability in bilateral primary motor cortex (M1) is elevated 300 ms after emotional stimulus onset (Borgomaneri, Gazzola & Avenanti, 2012; Borgomaneri, Gazzola & Avenanti, 2015).

**Figure 1:**
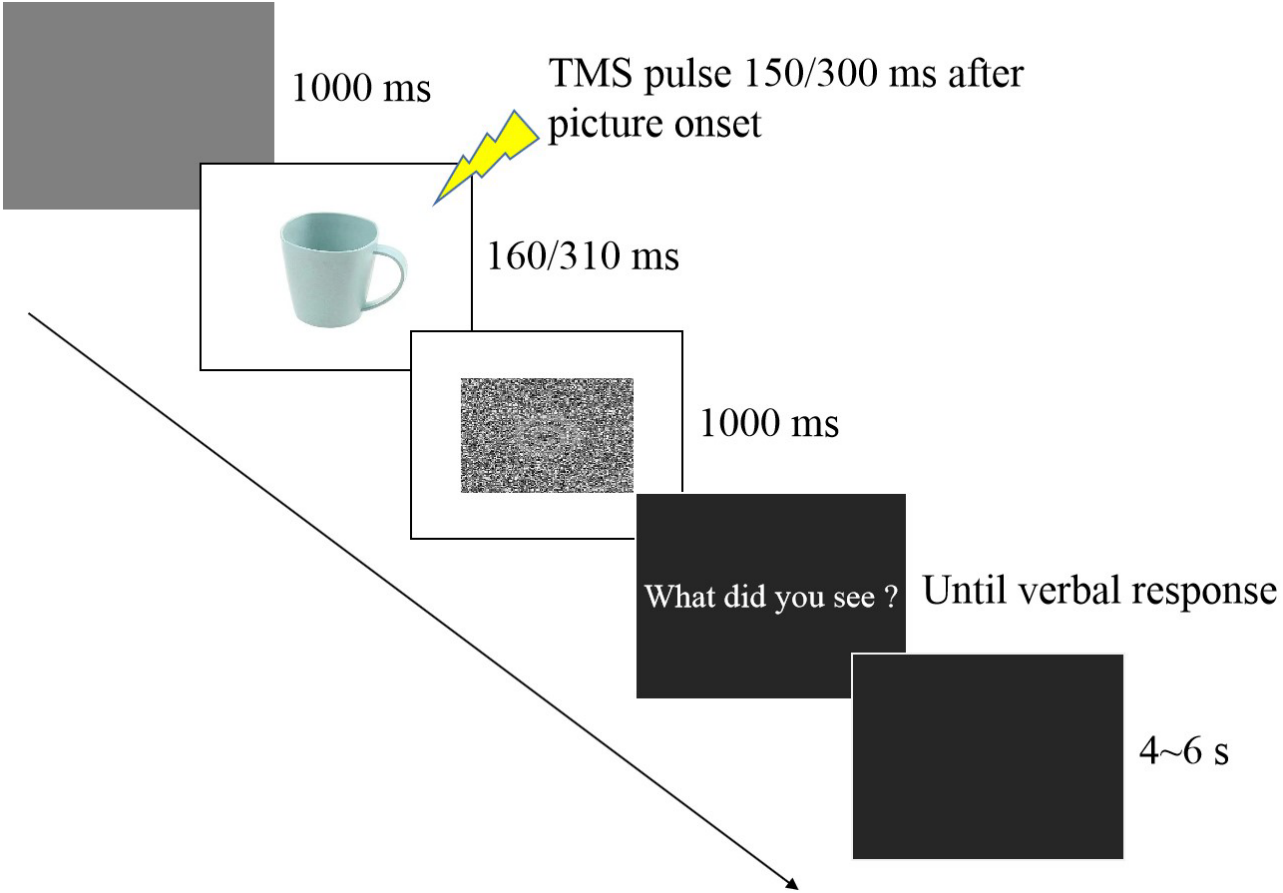
Trial sequence.

In the experimental session (see Fig. 1), participants were seated in a quiet room at approximately 50 cm from the computer screen and performed an emotion recognition task consisting of 60 trials, with 10 trials for each valence (negative, neutral, positive) at each time point (150 ms and 300 ms). Each stimulus was only presented once. Each experimental trial was initiated by a 1,000 ms presentation of a blank grey screen. Subsequently, a stimulus image was presented randomly, followed by a random-dot mask presented for 1,000 ms. Stimulus duration included two conditions (160 ms and 310 ms) that were equally and randomly distributed in the experimental session. TMS was delivered at 150 ms (160 ms stimulus duration) or 300 ms (310 ms duration) from stimulus onset after which the question “What did you see?” appeared on the screen, and the participant provided a verbal response to classify the picture as negative, neutral, or positive. An experimenter recorded the answer by pressing a computer key. To avoid changes in excitability due to verbal response (Tokimura et al., 1996), participants were asked to answer approximately 2–3 s after the TMS pulse (Tidoni et al., 2013). After the response, the screen appeared black for 4–6 s, ensuring an inter-pulse interval greater than 10 s and thereby avoiding changes in motor excitability due to TMS per se (Chen et al., 1997) which was directly confirmed by the absence of changes in MEP amplitudes between the two baseline blocks before and after the experimental session (*p* = 0.843).

After TMS, each participant was presented with all of the stimuli in a randomized order and asked to judge arousal and valence on a scale ranging from one (very calm for arousal and extremely negative for valence) to nine (very excited for arousal and extremely positive for valence).

### Data analysis

The rating of valence and arousal for each type of pictures (negative, neutral, and positive) was first averaged for each participant. Mean ratings for arousal and valence were not normally distributed and thus were analyzed by means of nonparametric Friedman ANOVAs and Bonferroni-corrected planned nonparametric comparisons.

Accuracy was computed for each valence (negative, neutral, positive) at each time point (150 ms and 300 ms) in each subject. To examine the effect of gender on emotion recognition, a four-way repeated-measures ANOVA was conducted for accuracy, with gender (male vs. female) and stimulated hemisphere (right M1 vs. left M1) as between-subject variables, and valence (negative, neutral, positive) and time point (150 ms vs. 300 ms) as within-subject variables.

The mean peak-to-peak MEP amplitude for each condition (negative, neutral, and positive) was expressed as a ratio of the mean peak-to-peak MEP amplitude at baseline (condition/baseline) (Xia et al., 2021). Trials were excluded from further analysis if voluntary root mean square EMG in the 100 ms before the TMS pulse exceeded two SD of the mean (2.63%) (Hannah et al., 2018), or if the response was incorrect (15.95%). The mean numbers of trials that were analyzed for each group are reported in Table S1. To examine the effect of gender on cortical excitability in bilateral M1 at the early and the later time points, a four-way repeated-measures ANOVA was conducted for MEP ratio at 150 ms or 300 ms, with gender (male vs. female) and stimulated hemisphere (right M1 vs. left M1) as between-subject variables, and valence (negative, neutral, positive) and time point as within-subject variables. Greenhouse-Geisser’s method was used to adjust for violation of sphericity. T-tests with Bonferroni corrections for multiple comparisons were used for post-hoc analysis if the ANOVA showed significant interactions. When the four-way repeated-measures ANOVA for a MEP ratio showed significant interactions, we compared the MEP ratios observed for positive, neutral, and negative pictures at each time point for each hemisphere in male subjects and in female subjects. We compared the hemispheric difference in MEP ratio for each valence at each time point in male subjects and in female subjects. Finally, we examined whether MEP ratios differed between the gender groups for each valence in each hemisphere and at each time point. *P*-values < 0.05 were considered significant. All statistical analyses were completed using the Statistical Package for Social Sciences software (SPSS version 25.0; Chicago, IL. USA).

## Results

### Behavioral performance in the emotion recognition task

The four-way ANOVA revealed a significant main effect of valence (*F* (2, 192) = 4.29, *p* = 0.015, *η*^2^ = 0.04) on accuracy. *Post hoc* tests confirmed that accuracy for negative pictures (87.40 ± 11.47%) was significantly higher than that for neutral pictures (82.75 ± 10.95%, *p* = 0.021, Cohen’s *d* = 0.41) and positive pictures (81.95 ± 16.44%, *p* = 0.023, Cohen’s *d* = 0.38), while no difference in accuracy was observed between neutral and positive pictures (*p* > 0.9, Cohen’s *d* = 0.06). There was also a significant main effect of time point (*F* (1, 96) = 41.87, *p* < 0.001, *η*^2^ = 0.30). A *post hoc* test revealed better performance at 300 ms (86.87 ± 7.40%) than that at 150 ms (81.20 ± 8.14%). There were no other significant main effects or interactions (all *p* > 0.079). Accuracy rates are shown in Table 1.

### Motor cortex excitability

Two-way ANOVAs with gender (male and female) and hemisphere (right M1 and left M1) as the between-subject factors were conducted on baseline MEP amplitude. No significant main effects on or interactions of baseline MEP amplitude were found (*p* ≥ 0.174). The baseline MEP amplitudes and mean MEP amplitudes are shown in Table 2.

A gender × hemisphere × valence × time point ANOVA for MEP ratio revealed a significant gender × time point interaction effect (*F* (1, 96) = 4.44, *p* = 0.038, *η*^2^ = 0.04), a significant valence × time point interaction effect (*F* (2, 192) = 4.65, *p* = 0.012, *η*^2^ = 0.05), and a significant gender × valence × time point interaction effect (*F* (2, 192) = 5.62, *p* = 0.005, *η*^2^ = 0.06).

Most importantly, a four-way interaction effect was also observed for MEP ratio (*F* (2, 192) = 4.31, *p* = 0.015, *η*^2^ = 0.04). *Post hoc* tests for the four-way interaction revealed that stimulation of the right M1 in male subjects lead to a significantly lower MEP ratio for positive pictures than for negative pictures (*p* = 0.043, Cohen’s *d* = 0.43) at the 150-ms time point, as well as a significantly higher MEP ratio for positive pictures than for negative (*p* = 0.001, Cohen’s *d* = 0.47) and neutral pictures (*p* < 0.001, Cohen’s *d* = 0.60) at the 300-ms time point. In male subjects, MEPs produced in response to right-M1 stimulation did not differ significantly between the positive- and neutral-picture conditions at 150 ms (*p* = 0. 832, Cohen’s *d* = 0.19), between the negative- and neutral-picture conditions at 150 ms (*p* = 0.158, Cohen’s *d* = 0.28), or between the negative- and neutral-picture conditions at 300 ms (*p* > 0.9, Cohen’s *d* = 0.13). MEPs produced in response to left-M1 stimulation did not differ significantly among the positive, neutral, and negative picture conditions in male subjects at either 150 ms or 300 ms (all *p* ≥ 0.448). Meanwhile, in female subjects, no significant differences among the positive, neutral, and negative picture conditions were observed for MEPs produced in response to stimulation of the left or right M1 at either 150 ms or 300 ms (all *p* ≥ 0.198).

**Table 1:** Demographic characteristics and behavioral data of the right and left M1 sessions at 150 ms and 300 ms in male and female subjects (M ± SD).

	Right M1		Left M1	
	Male	Female	Male	Female
Number	25	25	25	25
Mean age (years)	21.85 ± 1.74	21.52 ± 2.6	22.19 ± 1.79	21.08 ± 2.73
150 ms				
Accuracy for negative pictures (%)	84.80 ± 13.27	87.20 ± 12.42	84.00 ± 13.54	84.00 ± 17.08
Accuracy for neutral pictures (%)	75.60 ± 16.09	77.60 ± 17.63	85.20 ± 12.62	82.40 ± 10.12
Accuracy for positive pictures (%)	77.60 ± 23.32	79.60 ± 20.51	76.80 ± 21.74	79.60 ± 18.14
300 ms				
Accuracy for negative pictures (%)	90.80 ± 14.98	87.60 ± 15.62	90.80 ± 11.52	90.00 ± 12.25
Accuracy for neutral pictures (%)	83.20 ± 11.45	85.20 ± 11.22	87.60 ± 13.00	85.20 ± 12.95
Accuracy for positive pictures (%)	84.00 ± 16.83	82.80 ± 13.70	87.20 ± 16.71	88.00 ± 15.55

**Table 2:** Baseline MEP amplitudes and mean MEP amplitudes for right and left M1 stimulation in male and female subjects at 150 ms and 300 ms (M ± SD).

	Right M1		Left M1	
	Male	Female	Male	Female
Baseline MEP	1.05 ± 0.53	1.34 ± 0.84	1.38 ± 0.79	1.24 ± 0.95
150 ms				
Negative	1.82 ± 1.41	1.72 ± 0.98	1.81 ± 1.00	1.48 ± 0.84
Neutral	1.53 ± 1.04	1.73 ± 0.89	1.68 ± 0.91	1.52 ± 0.89
Positive	1.41 ± 0.99	1.67 ± 0.95	1.61 ± 0.92	1.45 ± 0.83
300 ms				
Negative	1.52 ± 1.13	1.64 ± 0.92	1.65 ± 1.01	1.50 ± 0.99
Neutral	1.36 ± 0.88	1.56 ± 1.02	1.68 ± 0.92	1.44 ± 1.07
Positive	1.89 ± 1.53	1.49 ± 0.98	1.74 ± 1.18	1.46 ± 0.91

When exposed to positive pictures, male subjects had a larger MEP ratio at 300 ms for trials in which the right M1 was stimulated than for trials in which the left M1 was stimulated (*p* = 0.005, Cohen’s *d* = 0.74), indicating that there was greater lateralization in motor excitability during affective processing than during emotionally neutral stimulus processing. In addition, when stimulation was applied to the right M1, males exhibited a larger MEP ratio than females at 300 ms in response to positive pictures (*p* = 0.001, Cohen’s *d* = 0.88).

**Figure 2:**
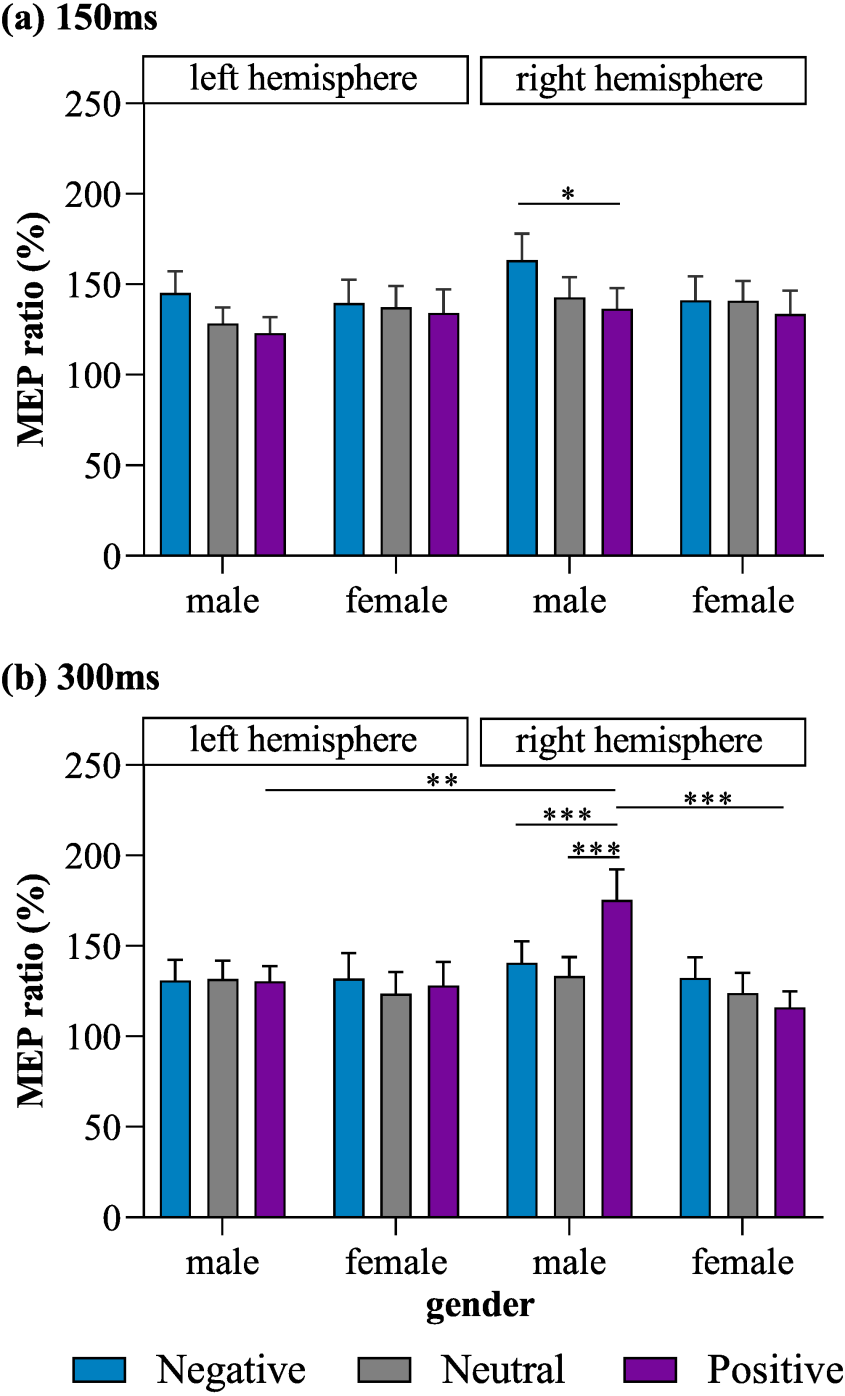
MEP ratios of each valence for left and right M1 stimulation in male and female subjects at (A) 150 ms and (B) 300 ms. *p < 0.05, **p < 0.01, ***p < 0.001.

No other significant main effects on or interactions of MEP ratios were found. The observed MEP ratios are illustrated in Fig. 2.

### Rating of Chinese Affective Picture System pictures

Table 3 shows the valence and arousal scores for negative, neutral, and positive pictures. Friedman ANOVAs for valence (*χ*^2^ = 208, *p* < 0.001) and arousal (*χ*^2^ = 163.01, *p* < 0.001) were both significant. Follow-up comparisons confirmed that valence of positive pictures was greater than neutral and negative pictures and valence of negative pictures was lower than that of neutral pictures (*p* < 0.001). Arousal was higher for positive and negative pictures relative to neutral pictures (*p* < 0.001).

## Discussion

The present study found gender and hemispheric differences in motor excitability during emotional processing through single-pulse TMS stimulation to the right and left M1.

### Rating of Chinese Affective Picture System pictures

It is widely accepted that negative stimuli require more intense and urgent processing and faster responses. Numerous studies have indicated that negative stimuli elicit more rapid and more prominent responses compared to neutral and positive stimuli (Borgomaneri, Gazzola & Avenanti, 2014; Carretié et al., 2009; Olofsson et al., 2008; Pourtois et al., 2004; Yuan et al., 2007). In agreement, the present study found significantly higher accuracy for negative pictures than for neutral and positive pictures during emotional processing. Behavioral performance was better 300 ms after stimulus onset than 150 ms after stimulus onset, which may have been due to 150 ms being too brief a period in which to classify a picture reliably.

**Table 3:** Self-report valence and arousal of the pictures.

	Negative pictures	Neutral pictures	Positive pictures
Valence	2.73 ± 0.61	4.76 ± 0.41	6.71 ± 0.6
Arousal	6.22 ± 1.17	2.87 ± 0.78	5.48 ± 1.23

### Motor negativity bias in men in the early phase of emotional processing

Electrophysiological studies have found that compared to positive and neutral stimuli, negative stimuli are associated with larger amplitudes and shorter ERP component latencies (Carretié et al., 2006; Carretié et al., 2004; Yuan et al., 2007), suggesting that salient threats may produce rapid and autonomic recruiting of attentional resources. This bias toward recruitment of resources in response to negative stimuli has been confirmed at the motor level (Borgomaneri, Gazzola & Avenanti, 2014; Komeilipoor et al., 2013), and it has been proposed that this bias may be an adaptive response associated with flight/fight typically elicited by aversive stimuli (Carretié et al., 2009). Using single-pulse TMS, Borgomaneri, Gazzola & Avenanti (2014) found an increase in motor excitability during the presentation of emotionally negative scenes compared to both positive and neutral scenes at 150 ms from stimulus onset, suggesting that emotionally negative events require motor reactions to be more immediately mobilized. The current findings expanded on these previous studies by demonstrating that the early negative bias is not limited to brain areas involved in emotion processing but extends to cortical motor representation. In addition, in the present study, we found that an increase in motor excitability in response to negative stimuli was observed only in male subjects. This finding suggests that emotionally negative stimuli may require a more vigorous and immediate motor reaction in men than in women. This vigorous and immediate reaction may reflect an adaptive neural mechanism that increases the likelihood of survival by facilitating rapid coping with the presentation of aversive or dangerous events (Borgomaneri, Gazzola & Avenanti, 2014; Carretié et al., 2006; Carretié et al., 2009). However, this result contradicts those of prior studies indicating that women tend to be more sensitive to emotionally negative information than men (Domes et al., 2010; Gard & Kring, 2007; Li, Yuan & Lin, 2008). It is possible that the negativity bias in women is observed at the level of perceptual processing and at the cortical level, such as activation of excitatory cortical interneurons (*i.e.*, intracortical facilitation), rather than being reflected at the level of corticospinal excitability (Borgomaneri, Francesca & Avenanti, 2017; Borgomaneri et al., 2015). Additionally, the negative pictures used in this study were negative situational pictures that evoke various emotions (threat/fear, unpleasantness, sadness). It is possible that stimuli associated with danger (such as threatening emotional scenes and fearful expressions) specifically facilitate the excitability of motor representations in women (Borgomaneri, Gazzola & Avenanti, 2014; Giovannelli et al., 2013; Schutter, Hofman & Honk, 2008). Gender differences in motor negativity bias have not been well studied. Future studies employing refined experimental materials in large samples are needed to probe gender differences in emotional stimulus-evoked motor cortex excitability.

### Motor positivity bias in men in a late phase of emotional processing

Stimulation to the right M1 lead to a significantly higher MEP ratio in response to positive pictures than in response to negative and neutral pictures in men at the longer latency time point (300 ms after stimulus onset). This result is consistent with previous studies reporting that men show a perceptual bias toward positive stimuli (Natale, Gur & Gur, 1983; Ran, 2018; Syrjänen & Wiens, 2013). This purported bias in emotional processing in men has been supported by neuroimaging studies showing greater brain activity in frontal regions and amygdala of men, compared to findings in women, during exposure to positive emotional photo stimuli (Fine, Semrud-Clikeman & Zhu, 2009; Stevens & Hamann, 2012; Wrase et al., 2003). Our results support a positivity bias in men at the motor level in that they show greater motor excitability when observing positive pictures relative to negative and neutral pictures. This motor positivity bias occurred in a late phase of emotional processing that coincides with the timing of electrophysiological measures of positive bias in men (Syrjänen & Wiens, 2013). Previous studies have shown that MEPs recorded 300 ms after stimulus onset increased in amplitude when emotional and neutral body stimuli were presented (Borgomaneri, Gazzola & Avenanti, 2012; Borgomaneri, Gazzola & Avenanti, 2015), supporting the notion that motor facilitation may depend more on the perceived motion implied by the observed action than on emotional valence. The inconsistency between these prior studies and the present study may be due, at least in part, to methodological differences. In the former studies, emotional body pictures were used as experimental materials and the processing of action information may have taken precedence over the processing of emotional valence. In a study in which they assessed motor excitability in subjects observing and categorizing positive, neutral, and negative scenes, Borgomaneri, Gazzola & Avenanti (2014) found increased MEPs for both positive and negative pictures, relative to MEPs for neutral pictures, consistent with increased reactivity to emotionally arousing scenes. The present study suggests that there may be emotion-specific modulation in the right hemisphere that is specialized for emotional processing in men. Future studies investigating motor excitability elicited by emotional stimuli should consider gender as a possible influencing factor.

### Rightward lateralization in men during emotional processing

When observing positive pictures, male subjects showed larger MEP ratios at the right M1 compared to the left M1 in the later temporal condition (300 ms). Similarly, when observing negative pictures, male subjects showed larger MEP ratios at the right M1 compared to the left M1 in the early temporal condition (150 ms). Together, these results suggest that male subjects show more lateralization in motor excitability during affective processing in both early and late emotional processing. Previous studies have suggested that males demonstrate more lateralization of brain function and cortical asymmetry than females in emotional processing (Killgore & Yurgelun-Todd, 2001; Wager et al., 2003). Some researchers have proposed that women tend to have more activity in left-hemispheric regions while men tend to show more right-hemispheric activity (Cahill et al., 2004; Canli et al., 2002). Furthermore, the direction of the hemispheric lateralization in males and females differs with valence, with happy faces producing greater right than left amygdala activation in males but not females (Killgore & Yurgelun-Todd, 2001). An ERP study also revealed greater neural responses in response to happy faces compared to angry faces in the right hemisphere in men (Ran, 2018). With regard to the excitability of the corticospinal motor tract, increased excitability has been shown to be lateralized as a function of stimulus valence (Komeilipoor et al., 2013; Tormos et al., 1997). For example, Komeilipoor et al. (2013) found that exposure to unpleasant sound stimuli resulted in a significantly higher facilitation of motor potentials evoked in the left hemisphere, while pleasant sound stimuli yielded a greater corticospinal motor tract excitability in the right hemisphere. Our findings are consistent with these previous studies and support an asymmetric modulation of motor excitability as a function of gender as well as emotional valence.

### Limitations

Our study has three notable limitations. First, we assessed left- and right-hemispheric motor excitability in separate individuals to minimize the practice effect. Individual differences between the two groups could have affected experimental results. Thus, importantly, we plan to investigate brain lateralization effects in future within-subject design studies. Second, because we explored motor excitability 150 ms and 300 ms after stimulus onset, we cannot exclude the possibility that emotion-specific stimuli may modulate motor excitability at non-observed time points. Third, we did not measure psychological characteristics (such as anxiety levels) in both groups, which may have influenced the results of this study. Future research could investigate the cortical excitability during processing emotional pictures in individuals with different anxiety levels. Fourth, the image presentation time is 6 ms longer than expected due to screen resolution issues. We will continue to update and improve the code to implement TMS and present images more accurately to solve this error.

## Conclusions

The results indicate a motor negativity bias in the early phase and a motor positivity bias in the later phase of emotional processing in men. Furthermore, there was a gender difference in laterality with a right hemisphere lateralization in males during emotional processing. The current study provides neurophysiological support for gender differences in motor excitability during emotional processing.

##  Supplemental Information

10.7717/peerj.13987/supp-1 Data S1 Raw data

10.7717/peerj.13987/supp-2 Table S1 Average number of trials that were analyzed for each group

10.7717/peerj.13987/supp-3 Supplemental Information 1 Codes for picture presentation time and TMS pulse start time

## Additional Information and Declarations

### Data Availability

The following information was supplied regarding data availability: The raw measurements are available in the Supplemental Files.
