# Supplementary material for: Gender dimorphic M1 excitability during emotional processing: a transcranial magnetic stimulation study
Source: PeerJ. 2022 Aug 30;10:e13987. doi: 10.7717/peerj.13987 (PMC9438768; doi:10.7717/peerj.13987)
Supplement: Table S1 [file peerj-10-13987-s002.docx]

|  | right M1 | |  | left M1 | |
| --- | --- | --- | --- | --- | --- |
|  | male | female |  | male | female |
| negative | 390 | 393 |  | 398 | 407 |
| neutral | 377 | 401 |  | 420 | 406 |
| positive | 423 | 426 |  | 427 | 417 |
